# 

**Published:** 1888-07

**Authors:** 


Chronic Diseases. E. J. L., . . . . . . . . . . . 335
Tracheotomy—A Plea for Natural Death. Stuart Close, M. D., . 340
Class-Room Talks, No. 6. Professor J. T. Kent, M. D., . . . 343
A Dietetic Case. A. McNeil, M. D., . . . . . . . . . 345
Dietetics: Its Bearing on Special and General Tissue Building. W. J. Thayer, D. D. S., M. D., . . . . . . . . . . 346
Proceedings of the Boston Organon Society. S. A. Kimball, M. D., *Secretary,* . . . . . . . . . . . . . 351
Proceedings of Syracuse Hahnemannian Club. Frederick Hooker, M. D., *Secretary,* . . . . . . . . . . . 357
Proceedings of Rochester Hahnemannian Society. W. H. Baker, M. D., *Secretary,* . . . . . . . . . . . . 360
Constipation and Some Prominent Remedies for its Treatment. Alexander C. Hermance, M. D., . . . . . . . . 364
The Vital Force. W. A. Hawley, M. D., . . . . . . . 368
Clinical Notes. E. W. M. Cameron, M. D., . . . . . 370
Rheumatism, and a Plea for Higher Potencies. Edward Rushmore, M. D., . . . . . . . . . . . . . . 372
Verifications of Symptoms. Flora A. Waddell, M. D., . . . 377
Sulphur in Melancholia. J. M. Dutton, M. D., . . . . . 380
Adhesions following Pelvic Peritonitis. J. M. Dutton, M. D., . . 382
Comparisons of Aconite with Apis and Gelsemium in Fever, . . 383
A Correction. S. L., . . . . . . . . . . . . 384
Queries as to Rhus and other Poisonings. Frederick Hooker, M. D., 384
Hahnemann's *Organon*—A Query. S. Lilienthal, M. D., . . . 385

### *BOOK NOTICES AND REVIEWS.*

Pathology, Diagnosis, and Treatment of Diseases of Women. By Graily Hewitt, M. D. New York, E. B. Treat & Co., . . . . 386
Publications of Massachusetts Homœopathic Medical Society, 1887, . 386
Physiology and Pathology of Diabetes. Prosper Bender, M. D., . 386
Address on Hospital and Dispensary Clinics. Prosper Bender M. D., . . . . . . . . . . . . . . . . 386
Croup and its Management. By Thomas Nichol, M. D. Montreal, W. Drysdale & Co., 1887, . . . . . . . . . . 386

